# Supplementary material for: Modeling glioblastoma heterogeneity as a dynamic network of cell states
Source: Mol Syst Biol. 2021 Sep 16;17(9):e10105. doi: 10.15252/msb.202010105 (PMC8444284; doi:10.15252/msb.202010105)
Supplement: Supplementary file 6 — Source Data for Figure 5 [file MSB-17-e10105-s004.zip › Figure5A_sourcedata/GSEA_3071/nosigdb_stateVI.GseaPreranked.1622795169056/SINGH_SOX2_UPREGULATED_TRANSCRIPTS_PMID_28122245.html]

Details for gene set SINGH\_SOX2\_UPREGULATED\_TRANSCRIPTS\_PMID\_28122245[GSEA]

|  || Dataset | state63071 |
| Phenotype | NoPhenotypeAvailable |
| Upregulated in class | na\_neg |
| GeneSet | SINGH\_SOX2\_UPREGULATED\_TRANSCRIPTS\_PMID\_28122245 |
| Enrichment Score (ES) | -0.5521152 |
| Normalized Enrichment Score (NES) | -2.960532 |
| Nominal p-value | 0.0 |
| FDR q-value | 0.0 |
| FWER p-Value | 0.0 |
Table: GSEA Results Summary

  

Fig 1: Enrichment plot: SINGH\_SOX2\_UPREGULATED\_TRANSCRIPTS\_PMID\_28122245      
 Profile of the Running ES Score & Positions of GeneSet Members on the Rank Ordered List

  

| PROBE | GENE SYMBOL | GENE\_TITLE | RANK IN GENE LIST | RANK METRIC SCORE | RUNNING ES | CORE ENRICHMENT || 1 | ADAMTS6 |  |  | 53 | 0.864 | -0.0091 | No |
| 2 | AJUBA |  |  | 154 | 0.659 | -0.0333 | No |
| 3 | MRPL52 |  |  | 206 | 0.598 | -0.0438 | No |
| 4 | CCDC80 |  |  | 343 | 0.514 | -0.0795 | No |
| 5 | PRMT5 |  |  | 372 | 0.502 | -0.0840 | No |
| 6 | NR3C1 |  |  | 395 | 0.492 | -0.0868 | No |
| 7 | SRP72 |  |  | 465 | 0.466 | -0.1035 | No |
| 8 | RPF1 |  |  | 523 | 0.449 | -0.1168 | No |
| 9 | PEF1 |  |  | 547 | 0.442 | -0.1202 | No |
| 10 | RIOK2 |  |  | 554 | 0.439 | -0.1188 | No |
| 11 | DPF3 |  |  | 601 | 0.425 | -0.1290 | No |
| 12 | HARS2 |  |  | 743 | 0.388 | -0.1671 | No |
| 13 | JAG1 |  |  | 876 | 0.365 | -0.2028 | No |
| 14 | ERCC2 |  |  | 949 | 0.353 | -0.2211 | No |
| 15 | PRR11 |  |  | 969 | 0.350 | -0.2241 | No |
| 16 | AIMP2 |  |  | 973 | 0.349 | -0.2224 | No |
| 17 | HIST1H2BD |  |  | 1045 | 0.338 | -0.2406 | No |
| 18 | CCND2 |  |  | 1064 | 0.335 | -0.2434 | No |
| 19 | TAF12 |  |  | 1083 | 0.333 | -0.2462 | No |
| 20 | TANGO6 |  |  | 1461 | 0.284 | -0.3535 | No |
| 21 | REPS1 |  |  | 1524 | 0.277 | -0.3695 | No |
| 22 | RBM8A |  |  | 1558 | 0.274 | -0.3771 | No |
| 23 | RAD23B |  |  | 1586 | 0.270 | -0.3830 | No |
| 24 | POLR3B |  |  | 1632 | 0.265 | -0.3941 | No |
| 25 | CTNNBL1 |  |  | 1690 | 0.261 | -0.4088 | No |
| 26 | ZBTB2 |  |  | 1722 | 0.258 | -0.4159 | No |
| 27 | RBM23 |  |  | 1824 | -0.252 | -0.4434 | No |
| 28 | RPA1 |  |  | 1849 | -0.256 | -0.4485 | No |
| 29 | COMMD2 |  |  | 1871 | -0.260 | -0.4527 | No |
| 30 | KIAA1958 |  |  | 1878 | -0.261 | -0.4525 | No |
| 31 | PHLPP1 |  |  | 1937 | -0.269 | -0.4674 | No |
| 32 | ZBTB10 |  |  | 1941 | -0.270 | -0.4663 | No |
| 33 | HIST1H3D |  |  | 1946 | -0.270 | -0.4655 | No |
| 34 | MPDZ |  |  | 1948 | -0.270 | -0.4638 | No |
| 35 | ARHGAP35 |  |  | 1964 | -0.273 | -0.4662 | No |
| 36 | OLIG2 |  |  | 1968 | -0.274 | -0.4651 | No |
| 37 | AURKB |  |  | 1978 | -0.274 | -0.4657 | No |
| 38 | STRADA |  |  | 2003 | -0.279 | -0.4707 | No |
| 39 | DUSP16 |  |  | 2009 | -0.280 | -0.4701 | No |
| 40 | SUCO |  |  | 2013 | -0.281 | -0.4689 | No |
| 41 | TNRC6C |  |  | 2040 | -0.284 | -0.4744 | No |
| 42 | SOX5 |  |  | 2064 | -0.289 | -0.4790 | No |
| 43 | FAM84B |  |  | 2080 | -0.291 | -0.4812 | No |
| 44 | FAM133B |  |  | 2098 | -0.295 | -0.4840 | No |
| 45 | GON4L |  |  | 2102 | -0.298 | -0.4827 | No |
| 46 | C1orf56 |  |  | 2115 | -0.300 | -0.4840 | No |
| 47 | FYN |  |  | 2118 | -0.301 | -0.4824 | No |
| 48 | SPAG9 |  |  | 2152 | -0.306 | -0.4898 | No |
| 49 | NPAS3 |  |  | 2184 | -0.312 | -0.4965 | No |
| 50 | UBALD2 |  |  | 2209 | -0.316 | -0.5012 | No |
| 51 | APBB2 |  |  | 2231 | -0.319 | -0.5050 | No |
| 52 | NHSL1 |  |  | 2240 | -0.322 | -0.5049 | No |
| 53 | CDH4 |  |  | 2262 | -0.326 | -0.5087 | No |
| 54 | TRAF4 |  |  | 2263 | -0.326 | -0.5063 | No |
| 55 | ZNF462 |  |  | 2279 | -0.330 | -0.5083 | No |
| 56 | APC |  |  | 2301 | -0.333 | -0.5119 | No |
| 57 | SFXN1 |  |  | 2316 | -0.336 | -0.5136 | No |
| 58 | ZNF608 |  |  | 2326 | -0.339 | -0.5137 | No |
| 59 | BMP7 |  |  | 2329 | -0.339 | -0.5118 | No |
| 60 | CTNND2 |  |  | 2331 | -0.339 | -0.5097 | No |
| 61 | PTK2 |  |  | 2347 | -0.343 | -0.5115 | No |
| 62 | BCL7A |  |  | 2359 | -0.346 | -0.5122 | No |
| 63 | EIF4G3 |  |  | 2364 | -0.348 | -0.5109 | No |
| 64 | FIGN |  |  | 2394 | -0.353 | -0.5167 | No |
| 65 | TBL1XR1 |  |  | 2402 | -0.353 | -0.5162 | No |
| 66 | HIST1H2AL |  |  | 2403 | -0.353 | -0.5136 | No |
| 67 | FANCI |  |  | 2411 | -0.356 | -0.5131 | No |
| 68 | RMI1 |  |  | 2432 | -0.362 | -0.5163 | No |
| 69 | KIF1B |  |  | 2443 | -0.365 | -0.5165 | No |
| 70 | GLCCI1 |  |  | 2521 | -0.383 | -0.5361 | No |
| 71 | KIF2A |  |  | 2532 | -0.385 | -0.5362 | No |
| 72 | ARHGAP42 |  |  | 2543 | -0.387 | -0.5363 | No |
| 73 | TMEM106C |  |  | 2554 | -0.389 | -0.5364 | No |
| 74 | VPS37B |  |  | 2565 | -0.391 | -0.5364 | No |
| 75 | SPRY2 |  |  | 2569 | -0.391 | -0.5345 | No |
| 76 | OSBPL6 |  |  | 2582 | -0.396 | -0.5351 | No |
| 77 | NDE1 |  |  | 2588 | -0.397 | -0.5336 | No |
| 78 | PBX3 |  |  | 2589 | -0.398 | -0.5307 | No |
| 79 | SP4 |  |  | 2613 | -0.404 | -0.5345 | No |
| 80 | FAT3 |  |  | 2625 | -0.406 | -0.5347 | No |
| 81 | PLCE1 |  |  | 2632 | -0.408 | -0.5335 | No |
| 82 | EXOC4 |  |  | 2669 | -0.415 | -0.5409 | No |
| 83 | SPEN |  |  | 2675 | -0.418 | -0.5394 | No |
| 84 | JAKMIP2 |  |  | 2716 | -0.430 | -0.5479 | No |
| 85 | PAG1 |  |  | 2720 | -0.430 | -0.5456 | No |
| 86 | KAT6A |  |  | 2723 | -0.431 | -0.5431 | No |
| 87 | AUTS2 |  |  | 2747 | -0.439 | -0.5465 | No |
| 88 | DHX40 |  |  | 2758 | -0.444 | -0.5462 | No |
| 89 | CDC45 |  |  | 2776 | -0.448 | -0.5479 | No |
| 90 | IPO9 |  |  | 2790 | -0.454 | -0.5484 | No |
| 91 | RAD21 |  |  | 2797 | -0.457 | -0.5468 | No |
| 92 | DOK5 |  |  | 2799 | -0.457 | -0.5438 | No |
| 93 | SMAD1 |  |  | 2815 | -0.461 | -0.5448 | No |
| 94 | POGZ |  |  | 2823 | -0.463 | -0.5435 | No |
| 95 | QKI |  |  | 2844 | -0.474 | -0.5458 | No |
| 96 | ZNF680 |  |  | 2861 | -0.482 | -0.5470 | No |
| 97 | UBE2T |  |  | 2868 | -0.484 | -0.5452 | No |
| 98 | SEMA6A |  |  | 2881 | -0.487 | -0.5452 | No |
| 99 | GLI3 |  |  | 2906 | -0.497 | -0.5485 | Yes |
| 100 | POU2F1 |  |  | 2908 | -0.498 | -0.5452 | Yes |
| 101 | ATF7IP |  |  | 2920 | -0.502 | -0.5447 | Yes |
| 102 | WDR82 |  |  | 2932 | -0.507 | -0.5442 | Yes |
| 103 | NCAPG |  |  | 2951 | -0.514 | -0.5457 | Yes |
| 104 | TRIL |  |  | 2958 | -0.519 | -0.5437 | Yes |
| 105 | SOX21 |  |  | 2979 | -0.529 | -0.5457 | Yes |
| 106 | MSI2 |  |  | 2992 | -0.537 | -0.5453 | Yes |
| 107 | SETD5 |  |  | 2993 | -0.537 | -0.5414 | Yes |
| 108 | SP3 |  |  | 2995 | -0.538 | -0.5378 | Yes |
| 109 | NES |  |  | 3001 | -0.542 | -0.5353 | Yes |
| 110 | TMPO |  |  | 3022 | -0.552 | -0.5371 | Yes |
| 111 | DCAF16 |  |  | 3043 | -0.560 | -0.5388 | Yes |
| 112 | FOXO3 |  |  | 3053 | -0.564 | -0.5373 | Yes |
| 113 | RFTN2 |  |  | 3065 | -0.570 | -0.5364 | Yes |
| 114 | SOX4 |  |  | 3066 | -0.571 | -0.5323 | Yes |
| 115 | PTPRA |  |  | 3073 | -0.576 | -0.5298 | Yes |
| 116 | SHC3 |  |  | 3093 | -0.581 | -0.5311 | Yes |
| 117 | RIMKLB |  |  | 3097 | -0.583 | -0.5278 | Yes |
| 118 | TRIM24 |  |  | 3106 | -0.590 | -0.5258 | Yes |
| 119 | PTOV1 |  |  | 3114 | -0.594 | -0.5235 | Yes |
| 120 | CELSR2 |  |  | 3121 | -0.598 | -0.5209 | Yes |
| 121 | OLIG1 |  |  | 3126 | -0.601 | -0.5177 | Yes |
| 122 | SCD5 |  |  | 3135 | -0.611 | -0.5156 | Yes |
| 123 | SPATA6 |  |  | 3143 | -0.614 | -0.5132 | Yes |
| 124 | ITGB8 |  |  | 3148 | -0.616 | -0.5099 | Yes |
| 125 | SUGP2 |  |  | 3150 | -0.618 | -0.5057 | Yes |
| 126 | SRI |  |  | 3179 | -0.643 | -0.5091 | Yes |
| 127 | PBX1 |  |  | 3200 | -0.664 | -0.5101 | Yes |
| 128 | CDK5RAP2 |  |  | 3205 | -0.672 | -0.5064 | Yes |
| 129 | USP1 |  |  | 3233 | -0.699 | -0.5092 | Yes |
| 130 | HIST1H2AH |  |  | 3274 | -0.734 | -0.5155 | Yes |
| 131 | SOX9 |  |  | 3280 | -0.739 | -0.5115 | Yes |
| 132 | EZH2 |  |  | 3282 | -0.741 | -0.5065 | Yes |
| 133 | SALL1 |  |  | 3284 | -0.742 | -0.5014 | Yes |
| 134 | STIM2 |  |  | 3308 | -0.772 | -0.5024 | Yes |
| 135 | SOX2 |  |  | 3314 | -0.778 | -0.4982 | Yes |
| 136 | MLC1 |  |  | 3326 | -0.793 | -0.4957 | Yes |
| 137 | BRCA1 |  |  | 3335 | -0.802 | -0.4922 | Yes |
| 138 | TNIK |  |  | 3355 | -0.822 | -0.4917 | Yes |
| 139 | ZEB1 |  |  | 3363 | -0.827 | -0.4878 | Yes |
| 140 | NLGN1 |  |  | 3364 | -0.831 | -0.4817 | Yes |
| 141 | ETV1 |  |  | 3378 | -0.857 | -0.4793 | Yes |
| 142 | WSCD1 |  |  | 3379 | -0.860 | -0.4730 | Yes |
| 143 | NKAIN3 |  |  | 3389 | -0.873 | -0.4693 | Yes |
| 144 | CDK1 |  |  | 3396 | -0.883 | -0.4646 | Yes |
| 145 | GAB1 |  |  | 3397 | -0.885 | -0.4582 | Yes |
| 146 | CTH |  |  | 3401 | -0.894 | -0.4526 | Yes |
| 147 | FBXO5 |  |  | 3413 | -0.919 | -0.4491 | Yes |
| 148 | ROBO2 |  |  | 3419 | -0.939 | -0.4438 | Yes |
| 149 | LDLRAD3 |  |  | 3424 | -0.946 | -0.4381 | Yes |
| 150 | CDKN1B |  |  | 3426 | -0.951 | -0.4314 | Yes |
| 151 | GOLM1 |  |  | 3435 | -0.972 | -0.4267 | Yes |
| 152 | TRIM9 |  |  | 3442 | -0.985 | -0.4213 | Yes |
| 153 | SOX11 |  |  | 3443 | -0.989 | -0.4141 | Yes |
| 154 | TUBA1A |  |  | 3447 | -1.002 | -0.4077 | Yes |
| 155 | ETV5 |  |  | 3466 | -1.043 | -0.4054 | Yes |
| 156 | KIF21A |  |  | 3482 | -1.070 | -0.4020 | Yes |
| 157 | LCORL |  |  | 3490 | -1.089 | -0.3961 | Yes |
| 158 | TCF12 |  |  | 3494 | -1.100 | -0.3890 | Yes |
| 159 | RGS12 |  |  | 3495 | -1.106 | -0.3810 | Yes |
| 160 | HIST1H1E |  |  | 3504 | -1.125 | -0.3751 | Yes |
| 161 | NFIB |  |  | 3508 | -1.145 | -0.3677 | Yes |
| 162 | HIST1H1C |  |  | 3509 | -1.145 | -0.3594 | Yes |
| 163 | NOVA1 |  |  | 3510 | -1.148 | -0.3510 | Yes |
| 164 | NCAM1 |  |  | 3514 | -1.168 | -0.3434 | Yes |
| 165 | GPM6A |  |  | 3515 | -1.169 | -0.3349 | Yes |
| 166 | SOX6 |  |  | 3517 | -1.174 | -0.3267 | Yes |
| 167 | DPYSL3 |  |  | 3521 | -1.188 | -0.3190 | Yes |
| 168 | SPECC1 |  |  | 3523 | -1.202 | -0.3105 | Yes |
| 169 | DCLK2 |  |  | 3524 | -1.206 | -0.3018 | Yes |
| 170 | TCF4 |  |  | 3528 | -1.214 | -0.2938 | Yes |
| 171 | TUBB2B |  |  | 3532 | -1.228 | -0.2858 | Yes |
| 172 | LSAMP |  |  | 3537 | -1.257 | -0.2778 | Yes |
| 173 | TTC28 |  |  | 3542 | -1.292 | -0.2696 | Yes |
| 174 | CDH2 |  |  | 3543 | -1.297 | -0.2602 | Yes |
| 175 | KAT6B |  |  | 3547 | -1.324 | -0.2515 | Yes |
| 176 | KLHDC8A |  |  | 3552 | -1.370 | -0.2427 | Yes |
| 177 | CDCA7L |  |  | 3557 | -1.393 | -0.2337 | Yes |
| 178 | ZBTB20 |  |  | 3567 | -1.435 | -0.2259 | Yes |
| 179 | LINC00461 |  |  | 3569 | -1.442 | -0.2158 | Yes |
| 180 | FAM181B |  |  | 3582 | -1.594 | -0.2077 | Yes |
| 181 | MAP3K1 |  |  | 3584 | -1.605 | -0.1963 | Yes |
| 182 | SYT11 |  |  | 3587 | -1.620 | -0.1851 | Yes |
| 183 | HIST1H1B |  |  | 3592 | -1.679 | -0.1741 | Yes |
| 184 | HIST1H1D |  |  | 3596 | -1.757 | -0.1622 | Yes |
| 185 | MAP2 |  |  | 3597 | -1.763 | -0.1494 | Yes |
| 186 | POU3F2 |  |  | 3610 | -1.933 | -0.1389 | Yes |
| 187 | SCRG1 |  |  | 3613 | -2.165 | -0.1238 | Yes |
| 188 | ID4 |  |  | 3620 | -2.343 | -0.1085 | Yes |
| 189 | NFIA |  |  | 3621 | -2.420 | -0.0909 | Yes |
| 190 | C1orf61 |  |  | 3626 | -2.616 | -0.0731 | Yes |
| 191 | PTN |  |  | 3632 | -2.826 | -0.0541 | Yes |
| 192 | LMO1 |  |  | 3635 | -3.371 | -0.0302 | Yes |
| 193 | PTPRZ1 |  |  | 3637 | -4.237 | 0.0003 | Yes |
Table: GSEA details [plain text format]

  

Fig 2: SINGH\_SOX2\_UPREGULATED\_TRANSCRIPTS\_PMID\_28122245: Random ES distribution      
 Gene set null distribution of ES for **SINGH\_SOX2\_UPREGULATED\_TRANSCRIPTS\_PMID\_28122245**
